# Disentangling Drivers of Meteorological Droughts in the European Greater Alpine Region During the Last Two Centuries

**DOI:** 10.1029/2018JD029527

**Published:** 2019-12-02

**Authors:** K. Haslinger, M. Hofstätter, C. Kroisleitner, W. Schöner, G. Laaha, F. Holawe, G. Blöschl

**Affiliations:** ^1^ Climate Research Department Central Institute for Meteorology and Geodynamics (ZAMG) Vienna Austria; ^2^ Institute for Hydraulic and Water Resources Engineering and Centre for Water Resource Systems Vienna University of Technology Vienna Austria; ^3^ b.geos GmbH Korneuburg Austria; ^4^ Department of Geography and Regional Sciences University of Graz Graz Austria; ^5^ Institute of Applied Statistics and Computing University of Natural Resources and Life Sciences (BOKU) Vienna Austria; ^6^ Department of Geography and Regional Research University of Vienna Vienna Austria

**Keywords:** drought, atmospheric circulation, circulation types, soil moisture preciptation feedback, Europe, climate change

## Abstract

This study investigates the atmospheric drivers of severe precipitation deficits in the Greater Alpine Region during the last 210 years utilizing a daily atmospheric circulation type reconstruction. Precipitation deficit tends to be higher during periods with more frequent anticyclonic (dry) and less frequent cyclonic (wet) circulation types, as would be expected. However, circulation characteristics are not the main drivers of summer precipitation deficit. Dry soils in the warm season tend to limit precipitation, which is particularly the case for circulation types that are sensitive to a soil moisture‐precipitation feedback. This mechanism is of specific relevance in explaining the major drought decades of the 1860s and 1940s. Both episodes show large negative precipitation anomalies in spring followed by increasing frequencies of circulation types sensitive to soil moisture precipitation feedbacks. The dry springs of the 1860s were likely caused by circulation characteristics that were quite different from those of recent decades as a consequence of the large spatial extent of Arctic sea ice at the end of the Little Ice Age. On the other hand, the dry springs of the 1940s developed under a persistent positive pressure anomaly across Western and Central Europe, triggered by positive sea surface temperatures in the western subtropical Atlantic.

## Key Points

- Winter and spring droughts are strongly related to the Eastern Atlantic‐Western Russia atmospheric variability pattern
- Summer droughts are enhanced by prevailing dry spring conditions through a positive soil moisture‐precipitation feedback
- The major dry episodes in the 1860s and 1940s are particularly driven by this feedback mechanism
- The dry springs of the 1860s were likely caused by unusual circulation regimes, synchronized with large spatial extents of Arctic sea ice at the end of the Little Ice Age
- The dry springs of the 1940s are due to positive sea surface temperature anomalies in the western subtropical Atlantic, which are related to a positive Eastern Atlantic‐Western Russia pattern

## 1 Introduction

A prolonged lack of precipitation may have serious impacts on various aspects of human society. Water supply, agriculture, hydro power production, and river navigation are heavily affected as the drought signal propagates through the hydrological cycle from the surface to the soils, rivers, lakes, and groundwater (Sheffield & Wood, 2011; Tallaksen, 2006; Van Loon et al., 2012).

While it is essential to better understand droughts in a changing climate, whether and why droughts in Europe have changed in the past centuries is still under debate. Some studies have identified an increase in drought frequency and severity (e.g., Dai, 2013) while others suggest no significant changes have occurred (Sheffield et al., 2012). Although there are efforts to reconcile the diverging results in trend estimates in recent decades (Trenberth et al., 2014), a big drawback is the focus of most studies on the post 1950 period, rarely putting these trends into the context of centennial climate variability. Latest research (Hanel et al., 2018; Haslinger & Blöschl, 2017; Moravec et al., 2019), however, started to investigate droughts on timescale of 200+ years. The Intergovernmental Panel on Climate Change (2012, SREX) concludes that due to inconsistent signals of the various drought indicators, no clear trends for Central Europe can be inferred. Yet, there is evidence for an intensification of the water cycle with global warming (Held & Soden, 2006; Huntington et al., 2018), which might involve an intensification of drought conditions, especially in the summer (JJA) when soil moisture‐precipitation feedbacks are strongest (Seneviratne et al., 2010).

Several studies investigated the atmospheric drivers of individual extreme drought events in Europe. Ionita et al. (2017) analyzed the severe JJA drought of 2015 (Laaha et al., 2017; Van Lanen et al., 2016), finding that this event was triggered and enhanced by four heat waves caused by persistent blocking events and a deflection of the Atlantic storm tracks toward the North. Similarly, the extreme precipitation deficits of the Iberian drought in 2004/2005 (García‐Herrera et al., 2007) and the devastating JJA drought of 2003 (Black et al., 2004) were caused by anticyclonic circulation in combination with a positive soil moisture‐precipitation feedback.

There have also been studies that jointly analyzed drought mechanisms for a number of events. Kingston et al. (2006) and Lavers et al. (2013) investigated precipitation and streamflow anomalies across Northern Europe and found that the North Atlantic Oscillation (NAO) is a principal driver, which implies dry conditions during its negative phase, particularly in the winter (DJF). On the other hand, Linderholm et al. (2009) found that the NAO also plays a significant role in the drought development of Northern Europe during JJA. These findings are in line with other studies that highlight the NAO phases as an important cause of dryness/wetness variability over Europe (Hannaford et al., 2011; López‐Moreno & Vicente‐Serrano, 2008; Vicente‐Serrano et al., 2016). Accordingly, Northern Europe tends to exhibit strong negative correlations between NAO and droughts, Southern Europe strong positive correlations, and there is a zone with weaker correlations in between, for example, in France (Giuntoli et al., 2013). In consequence the NAO appears as a poor indicator for explaining wet or dry period occurrence in Central Europe, including the European Alps. However, divergent variants in terms of the definition of the NAO are usually utilized in those studies. Some use simple air pressure differences between stations in Iceland and Southern Europe (Jones et al., 1997), others the leading empirical orthogonal function mode of sea level pressure across Europe and the North Atlantic (Hurrell et al., 2003). Caution is needed when interpreting results, which may be contradictory, but are rooted in the usage of different NAO indices (see Hurrell & Deser, 2009) on one hand and on the nonstationarity of the NAO/drought relation (López‐Moreno & Vicente‐Serrano, 2008) on the other. Other large scale circulation indices explaining European climate variability include the Arctic Oscillation (Thompson & Wallace, 1998), which is strongly interrelated with the NAO, the Scandinavian Pattern (Bueh & Nakamura, 2007), the El Niño Southern Oscillation (Brönnimann, 2007), and the East Atlantic/Western Russia Pattern (EAWR, introduced by Barnston & Livezey, 1987 as the Eurasian Pattern type 2). The latter is related to drought‐inducing atmospheric circulation, as shown by Ionita (2014) who concluded that mid‐DJF to late spring (MAM) precipitation is strongly impacted by the EAWR teleconnection. Similarly, Kingston et al. (2015) found that while the NAO is an important driver of Northern European droughts, the EAWR is more important for other regions.

Apart from large‐scale modes as popular indices to link atmospheric circulation to droughts as described above, atmospheric circulation type (CT) classifications provide more detailed information on synoptic characteristics. This is on one hand due to the higher temporal resolution (usually daily or sub‐daily) and on the other hand because of the optimization of the classification scheme for a specific region. These features enables an even more detailed investigation of atmospheric processes during drought episodes, however, literature is sparse in this respect. The potential of using CTs in understanding hydrological drought development is shown for example by Fleig et al. (2010) for two case studies in the United Kingdom and Denmark. Furthermore, Beck et al. (2015) analyzed Standardized Precipitation Index variations on a 3‐month aggregation time scale in Germany and optimized a statistical downscaling model for reconstructing the Standardized Precipitation Index based on different CT configurations. Similarly, Vicente‐Serrano and López‐Moreno (2006) investigated DJF droughts in Northern Spain, their relation to weather types, and their connection to the NAO. However, all these examples mentioned here use post 1950 data. Just recently in a study for the United Kingdom (Richardson et al., 2018) data going back to 1850 investigated precipitation and drought variability in relation to weather types using different classification schemes.

Refocusing on literature considering the Alpine region in specific, Efthymiadis et al. (2007) found precipitation variability in the Southeastern Greater Alpine Region (GAR) to be forced by the NAO, while the EAWR was more important for the Northwestern GAR. Yet the NAO influence seems to vary considerably over time (Brunetti et al., 2006). Scherrer et al. (2016) showed the NAO to be of little influence for precipitation in Switzerland, the Eastern Atlantic blocking pattern to be important for the northern slopes of the Alps, and the Eastern Atlantic pattern to be relevant for the southern slopes. The specific atmospheric processes apart from large scale circulation modes forcing the development of drought conditions in the Greater Alpine Region, however, are not fully understood.

Moreover, the decades of the 1860s and 1940s have been highlighted in the literature as decades with exceptional drought conditions in the GAR. In a joint space‐time assessment, Haslinger and Blöschl (2017) found that these time periods show the highest intensities of precipitation deficit. These results are confirmed by van der Schrier et al. (2007) who analyzed soil moisture variability using the Palmer drought severity index and concluded that the time periods from the 1850s to the 1870s and the 1940s to the early 1950s stand out as persistent and exceptionally dry periods. Similarly, a hydrological analysis found these to be outstanding decades of streamflow anomalies in Central Europe (Pekarova, Miklanek, & Pekar, 2006). However, so far, little is known on the drivers of the 1860s and 1940s anomalies.

Given the existing literature there is a substantial research gap considering drought development on a long‐term perspective in the European Alpine Region. The general aim of this paper is therefore to investigate a range of atmospheric processes leading to severe drought events in the GAR through a wide assessment of the atmospheric processes and not just taking one specific potential driver (e.g., circulation) into account. With regard to getting a most comprehensive assessment of atmospheric drought development in the GAR in the past 200 years, the following specific aims are considered:

1. quantifying the relationship between precipitation deficit during drought events and atmospheric circulation anomalies using latest high quality CT reconstructions and furthermore relating those anomalies to large scale modes of variability (NAO, EAWR …);
2. evaluating seasonal differences between atmospheric forcing and soil moisture feedback by relating precipitation distributions to preceding soil moisture conditions with regard to differences in atmospheric circulation;
3. understanding the atmospheric drivers of the exceptionally dry decades of the 1860s and 1940s by analyzing the similarities and differences of those decades in terms of atmospheric circulation anomalies over the Alpine Region, the embedment in a large scale climate context, and emerging soil moisture precipitation feedback processes; and
4. discussing these in the context of possible future climate change.

We address these aims by making use of recently published data on the space‐time extent of meteorological drought events in the GAR back to 1801 (Haslinger & Blöschl, 2017) and a reconstruction of daily weather types back to 1763 tailored to precipitation in the Alpine region (Schwander et al., 2017), thus going significantly beyond existing research. A subset of this dataset spanning the 1801–2009 period is used in the current study.

## 2 Data

### 2.1 Drought Events

We use a subset of the Haslinger & Blöschl, (2017, HB17 hereafter) data set on meteorological drought events in the GAR. The data set consists of a total of 663 events; we only use the top 5% (34 events) in terms of their drought severity. Table 1 gives the main event characteristics, including dimensionless mean drought intensity and severity as derived by HB17. These were calculated by assessing the extent of spatially contiguous precipitation anomalies (nonexceedance of the 0.2 quantile) tracked along time to detect space‐time drought regions. The magnitude of the deviation below the 0.2 quantile at each grid point, identified as a drought area, is multiplied by the number of grid points with a respective area, yielding a drought intensity measure for each time step. Severity is given by the sum of all intensities during an event. The mean drought intensity of an event is the severity divided by the event duration (see HB17 for details). The corresponding spatial average over the GAR of precipitation, precipitation deficit (RR deficit thereafter), and accumulated precipitation deficit are also shown. The RR deficit is the difference between RR during the event and the climatological mean (1801–2010) precipitation. The accumulated precipitation deficit is the RR deficit multiplied by the duration. The drought duration as given by HB17 is based on centered 3‐month moving averages of precipitation. They determined the beginning of an event by the time step of detecting the first drought area. This means that the first time step includes information from the previous as well as the subsequent time steps. It is therefore necessary to consider the month before the first time step as well as the month after the last time step of HB17 events for a full assessment of the overall event precipitation deficit. The seasonal assignment is based on the mean drought intensity of the cold season (NDJFMA) and the warm season (MJJASO), where events are assigned to the seasons with higher mean drought intensity. The peak is defined as the month with the highest drought intensity. When interpreting the results of the HB17 drought event assessment, uncertainties have to be kept in mind. The gridded precipitation data set that they are based on (Efthymiadis et al., 2006) is using station data, which is of course sparse from the beginning of the 19th century. However, a comprehensive skill analysis of the station reconstruction and gridding procedure indicates robustness of the data from 1850 onwards. During the first decades of the 19th century larger uncertainties remain and have to be considered when interpreting the results of this paper.

**Table 1:** Drought Event Characteristics

Event	Date	Duration (months)	Season	Peak	Mean drought intensity	Drought severity	Average precipitation	Average precipitation deficit	Accumulated precipitation deficit
							(mm/d)	(mm/d)	(mm)
1822	January 1822–August 1822	8	cold	March 1822	776	4,655	2.27	−0.53	−127
1832	April 1832–November 1832	8	warm	September 1832	765	4,589	2.53	−0.69	−167
1834	February 1834–July 1834	6	cold	March 1834	1,679	6,715	1.92	−0.94	−169
1835	July 1834–February 1835	8	cold	October 1834	858	5,145	2.25	−0.70	−167
1852	December 1851–July 1852	8	cold	April 1852	1,027	6,159	2.08	−0.67	−162
1854	October 1853–June 1854	9	cold	March 1854	952	6,666	2.40	−0.46	−123
1858	September 1857–Apr 1857	8	cold	January 1858	1,234	7,403	2.00	−0.81	−195
1861	November 1860–December 1861	14	warm	September 1861	652	7,819	2.48	−0.48	−201
1865	February 1865–July 1865	6	warm	May 1865	1,573	6,291	1.97	−0.89	−161
1866	July 1865–February 1866	8	cold	December1866	864	5,186	2.35	−0.60	−144
1870	December 1869–September 1870	10	warm	April 1870	1,040	8,322	2.40	−0.44	−131
1872	July 1871–May 1872	11	cold	September 1871	553	4,977	2.52	−0.37	−121
1874	October 1873–June 1874	9	cold	January 1874	855	5,985	2.36	−0.50	−134
1880	October 1879–March 1880	6	cold	February 1880	1,180	4,719	1.68	−1.05	−188
1882	October 1881–June 1882	9	cold	December1881	1,074	7,517	2.16	−0.70	−188
1884	April 1883–September 1884	18	cold	February 1884	429	6,856	2.65	−0.37	−201
1890	November 1889–April 1890	6	cold	January 1890	1,119	4,477	1.87	−0.77	−139
1898	September 1897–March 1898	7	cold	December1897	882	4,412	2.42	−0.39	−81
1909	August 1908–May 1909	10	cold	November 1908	727	5,814	2.23	−0.64	−191
1921	August 1920–February 1922	19	cold	October 1921	977	16,610	2.24	−0.69	−394
1925	September 1924–March 1925	7	cold	December1924	1,121	5,605	2.18	−0.63	−133
1946	July 1945–January 1947	19	warm	Apr 1946	438	7,442	2.55	−0.42	−239
1947	April 1947–November 1947	8	warm	September 1947	1,130	6,777	2.38	−0.84	−202
1949	September 1948–Apr 1949	8	cold	January 1949	1,419	8,513	1.79	−1.02	−244
1952	February 1952–September 1952	8	warm	June 1952	855	5,130	2.56	−0.38	−90
1953	August 1953–February 1954	7	cold	November 1953	1,197	5,984	2.16	−0.77	−161
1962	May 1962–January 1963	9	warm	July 1962	920	6,442	2.65	−0.43	−117
1971	April 1971–December1971	9	warm	September 1971	840	5,877	2.42	−0.73	−196
1976	December1975–August 1976	9	warm	May 1976	773	5,410	2.22	−0.57	−155
1985	July 1985–December1985	6	cold	September 1985	1,299	5,197	2.27	−0.90	−163
1989	July 1988–March 1989	9	cold	December1988	729	5,106	2.27	−0.60	−161
1990	July 1989–March 1990	9	cold	November 1989	1,037	7,258	2.36	−0.51	−137
1993	November 1992–July 1993	9	cold	February 1993	830	5,808	2.46	−0.37	−101
2003	January 2003–September 2003	9	warm	March 2003	1,535	1,0742	1.87	−0.99	−268

*Note*. Mean drought intensity and drought severity are dimensionless measures of space‐time precipitation anomalies (see Haslinger & Blöschl, 2017), where drought severity is mean drought intensity times the duration; average precipitation is the observed precipitation averaged over the Greater Alpine Region divided by the duration in days, average precipitation deficit is the average precipitation divided by the climatological mean for the months under consideration; accumulated deficit is the average precipitation deficit times the duration in days.

### 2.2 Circulation Types

The relationship between droughts in the GAR and continental scale atmospheric weather patterns is investigated by use of the CT classification CAP7 (Schwander et al., 2017). CTs are a limited number of representative, stationary patterns of the continuum of the atmospheric circulation, and are frequently used to investigate their relationship with surface climate variables (Philipp et al., 2010). The choice of a certain CT classification depends on the parameter of interest, the number of classes defined therein, as well as on the classification method and the spatial domain (Beck & Philipp, 2010; Philipp et al., 2016).

For this study CAP7 (Schwander et al., 2017) is applied, which was specifically developed for the Central European region (Schwander et al., 2017, Figure 1 therein). The classification contains seven CTs, derived by principal component analysis in the reference period 1960–2000, followed by temporal clustering. A reconstruction of CAP7 in daily resolution is available back to the year 1763, whereas the subset for the 1801–2009 period is used in the current study.

**Figure 1:**
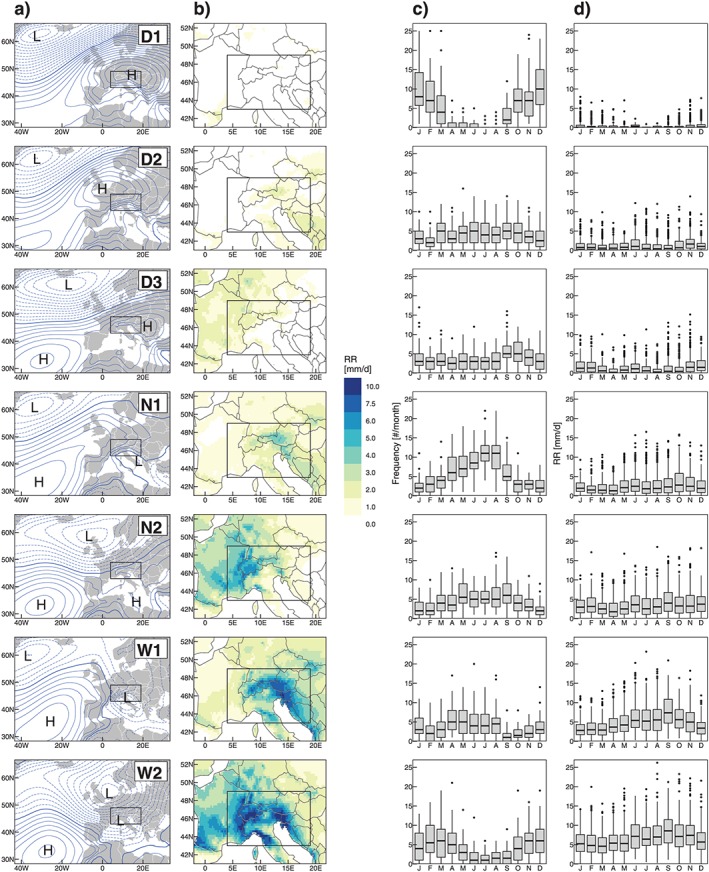
Characteristics of the (Schwander et al., 2017) circulation types: (a) mean sea level pressure based on 20CR, 1950–2009, (b) average precipitation based on E‐OBS data, 1950–2009, (c) monthly frequency (days per month), and (d) mean daily precipitation sum averaged over the Greater Alpine Region based on E‐OBS data, 1950–2009. The boxplots are constructed using the 0.25 and 0.75 quantiles for the bounding box, and the upper whisker extends from the bounding box to the highest value that is within 1.5 times the interquartile range; dots represent outliers beyond this range.

Figure 1 shows the main characteristics of the CTs. The original CT labels of 1–3, 4–5, and 6–7 of Schwander et al. (2017) has been changed to D1, D2, and D3 for the dry CTs with anticyclonic dominance over Central Europe, N1 and N2 for the neutral (weak pressure gradient) CTs, and W1 and W2 for the wet CTs, respectively (cf. Table 2). The average mean sea level pressure (Figure 1a) clearly indicates the anticyclonic flow over Central Europe for the three dry CTs (D1, D2, and D3), with a different location of the anticyclone center in each case. N1 exhibits a prevailing Northeasterly flow, however, with weak pressure gradients, induced by low pressure systems over the central and eastern Mediterranean and high pressure over the eastern Atlantic. In contrast, a more zonally (east‐west) flow is dominant during N2 with low pressure over the British Isles and high pressure in the eastern Balkans. The two wet CTs exhibit widespread low pressure directly over Central Europe. W1 shows prevailing meridional, northerly flows while W2 shows more zonal, westerly flows south of the Alps. In addition, the CTs are classified considering their large scale flow. D1 and W2 exhibit no large‐scale flow (anticyclonic/cyclonic center over Central Europe); D3 and N2 are predominantly under S/W flow, which means oceanic influence (Atlantic, Mediterranean), and D2, N1. and W1 show N/E flow, indicating continental influence.

**Table 2:** Characteristics of the (Schwander et al., 2017) Circulation Types

Abbreviation	Synoptic characteristics	Cyclonic/anticyclonic	Prevailing flow	Average RR (mm/d)	Large‐scale flow
D1	High pressure over Central Europe	Anticyclonic	—	0.5	—
D2	Indifferent Easterly flow	Anticyclonic	E	1.3	N/E
D3	Westerly flow over Northern Europe	Anticyclonic	W	1.4	S/W
N1	Indifferent Northeasterly flow	Indifferent	NE	2.7	N/E
N2	West‐southwesterly flow, cyclonic, flat pressure	Indifferent	WSW	3.6	S/W
W1	Northerly flow, cyclonic	Cyclonic	N	4.9	N/E
W2	Westerly flow over Southern Europe, cyclonic	Cyclonic	—	6.4	—

Abbreviation: RR = average precipitation.

Precipitation and Temperature‐Gridded Observations

The RR, RR deficit, and accumulated precipitation deficit per drought event in Table 1 is derived from the GAR‐gridded monthly precipitation data set (Efthymiadis et al., 2006), which covers the period 1801–2010 (updated by HB17) on a grid resolution of 10 arc minutes (~16 km). Air temperature is derived from an updated version of the GAR‐gridded monthly temperature data set (Chimani et al., 2013) for the period 1801–2010. In addition gridded daily mean temperature and precipitation data from the E‐OBS database (Haylock et al., 2008) is used for 1950–2009. A comparison of monthly precipitation averages over the GAR domain revealed differences between the GAR and the E‐OBS dataset (between −1.3% in January and −11.7% in July for the period 1950–2009), which is most likely due to differences in the station density, input data quality, and interpolation method. Therefore, a bias correction of both temperature and precipitation is necessary. We consider the GAR data as the reference since it served as the basis for the drought event detection by HB17. In a first step the E‐OBS data was bilinearly interpolated onto the GAR grid. Next we added a correction term to the daily temperature grids and applied a multiplicative correction term to the daily precipitation grids. These correction terms were estimated from the monthly biases of the E‐OBS precipitation totals and temperature means with respect to the GAR data for the 1950–2009 period.

### 2.3 Atmospheric Reanalysis and Climate Indices

Furthermore, atmospheric data (500‐hPa geopotential height, mean sea level pressure, and 500 and 300‐hPa wind speed as an indicator for the jet stream location) is used from the 20th Century Reanalysis project (20CR, Compo et al., 2011) from 1851 to 2009 over the region 80°W–60°E and 28°N–72°N. In addition, the NOAA Extended Reconstructed Sea Surface Temperature (SST) V4 (Huang et al., 2015) from 1854 to 2009 covering the North Atlantic and the Mediterranean (100°W–40°E and 25°N–75°N) is used. Finally, we used three atmospheric and oceanic circulation indices: the monthly NAO (Jones et al., 1997) from 1821 to 2009, a reconstruction of the monthly EAWR (Poirier et al., 2017) from 1801 to 2009, and the monthly Atlantic Multidecadal Oscillation Index (AMO, Enfield et al., 2001) from 1856 to 2009. The specific application of these data sets is explained in the section 3.

## 3 Methods

### 3.1 Frequency Anomaly of Anticyclonic CTs and Precipitation Efficiency

The analysis in this paper is based on two main variables. First the frequency anomaly of anticyclonic CTs (ACTs) for every single drought event is given by

(1) $$\text{FACT}=\frac{f_{\text{event}}}{f_{\text{clim}}}$$

where *FACT* is the frequency anomaly of ACTs (D1, D2, and D3) for a particular drought event; *f*_event_ is the observed frequency of ACTs during that event and *f*_clim_ is the long‐term (1801–2009) climatological mean frequency of ACTs; and *f*_clim_ is consequently calculated for all the months over which a given event extends. Values above (below) unity indicate more (fewer) days with ACTs than the climatological mean.

Secondly precipitation efficiency (*RR*_eff_) of a day with respect to the CT *c* of the day is defined as

(2) $${\mathrm{RR}}_{\mathrm{eff}}=\frac{{\mathrm{RR}}_{\mathrm{OBS}}}{\bar{RR_{\mathrm{CT}}}}$$

(3) $$\bar{RR_{\mathrm{CT}}}\left(c,m\right)=\frac{1}{n}\;\sum_{i=1}^{n}\mathrm{RR}\left(c,m\right)\;c\in \left[1;7\right],\;m\in \left[1;12\right]$$

where *RR*_OBS_ is the observed RR over the GAR on a given day, and $\bar{RR_{\mathrm{CT}}}$ is the mean average daily precipitation over the GAR for the particular CT *c* during the respective month *m* of the year. Values above (below) unity indicate more (less) precipitation on that particular day than expected from the climatological mean for the month of year and CT.

$\bar{RR_{\mathrm{CT}}}$ is calculated from the bias‐corrected daily E‐OBS fields (1950–2009) using equation (3), where *n* is the number of times CT *c* is observed in month *m* during the 1950–2009 period, and *RR*(*c*,*m*) is the respective precipitation on that days. RR efficiency could also be assessed on a daily basis where daily precipitation data is available, by relating the observed precipitation to the mean precipitation expected for the given day and occurring CT. However, in this paper RR efficiency is mostly considered as an average over the whole duration of a given event, denoted as the mean event RR efficiency. Only for the analysis of preceding soil moisture conditions during JJA for the years 1950–2009, RR efficiency is calculated on a daily basis using E‐OBS data.

The relationships between RR deficit during drought events, frequency anomaly of ACTs, and large‐scale atmospheric and oceanic indices are assessed by simple and multiple ordinary least squares linear regression models (cf. Wilks, 2011) on an event basis as well as on a seasonal basis (winter, spring, summer, autumn). Therefore, only subsets of those events are considered covering the respective season entirely (e.g., for an event stretching from January to May, MAM is used for the seasonal assessment as it is fully covered [March–April–May], DJF is not considered, since December is not a drought month). This means that event characteristics (RR deficit and frequency anomaly of ACTs) are split into seasonal averages and serve as data points for the regression.

To better understand the interrelationships between the two large scale circulation patterns (NAO and EAWR) and the emergence of distinct CTs, large‐scale atmospheric flow patterns for specific NAO and EAWR conditions are analyzed. Therefore, only those months where analyzed when the NAO index and/or the EAWR index is below (NAO− and EAWR−) or above (NAO+ and EAWR+) one standard deviation, leading to composite maps of z500 anomalies and jet stream wind speed. Furthermore, the frequency anomalies of CTs are analyzed in terms of their distributions during different NAO/EAWR conditions. The Kolmogorov‐Smirnov Test is applied to test for significant deviations of the frequency anomaly distribution. In addition, z500 and sea level pressure anomalies are calculated for assessing anomalies of the atmospheric circulation during selected time periods (e.g., drought decades of the 1860s and 1940s).

### 3.2 Analysis of Soil Moisture/SST Precipitation Coupling

To understand the potential coupling between soil moisture and precipitation we analyze precipitation efficiency in JJA as a function of the circulation characteristics and the soil moisture conditions. We used the climatic water balance (CWB) on a 3‐month (90 days, right sided) time scale averaged over the GAR as a large scale proxy for soil moisture conditions (Herold et al., 2016; Mueller & Seneviratne, 2012; Whan et al., 2015). The CWB is calculated from the E‐OBS daily fields of precipitation and a Hargreaves estimate of potential evapotranspiration from minimum and maximum air temperature (Hargreaves, 1975; Hargreaves & Allen, 2003) for the time period 1950–2009. For every day‐of‐year during the JJA months (92 days in total) the 10th percentile of the CWB is determined from the empirical cumulative distribution function. By extracting days where the CWB is below this threshold we retrieve those where the CWB is in an extremely dry state compared to the long‐term (1950–2009) mean. The analysis is then carried out by comparing the distributions of RR efficiency using the Kolmogorov‐Smirnov test to assess significant differences among CTs under dry and non‐dry conditions.

In addition, RR efficiency is also assessed in relation of SST anomalies in the Atlantic and the North Sea (20° W–10° E and 40° N–60° N) by comparing precipitation efficiency among CTs with regard to positive (>+1 °C) and negative (<−1 °C) SST anomalies in the given area.

### 3.3 Analysis of the Drought Decades of the 1860s and 1940s

The outstanding drought decades of the 1860s and 1940s have previously been explored either with a meteorological drought focus (Briffa, van der Schrier, & Jones, 2009; Brunetti et al., 2006; Haslinger & Blöschl, 2017; van der Schrier et al., 2007) or a hydrological focus (Pekarova et al., 2006) with less emphasis on the atmospheric drivers. For assessing the characteristics of these major drought episodes we do not only examine the detected drought events but also the wet and dry tail distribution of precipitation. This is done by first estimating the seasonal 0.2 (RRq20) and 0.8 (RRq80) precipitation quantiles from the empirical cumulative distribution of the entire time period (1801–2010). Then the seasonal 0.2 and 0.8 quantiles are estimated for every decade (e.g., 1801–1810). The anomaly is the relative deviation of the seasonal quantiles of a decade from the long‐term (1801–2010) quantiles of the respective season, RRq20 anomaly and RRq80 anomaly.

## 4 Results

### 4.1 Drought Driver #1: Atmospheric Circulation

**Figure 2:**
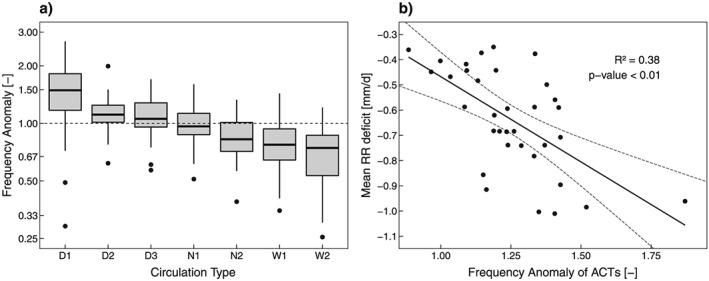
(a) Circulation type frequency anomalies (number of circulation type occurrences with respect to the long‐term mean 1801–2010) of individual drought events and (b) linear regression of frequency anomaly of ACTs (D1, D2, and D3) with respect to the long‐term mean against average prcipitation deficit during drought events; the dashed lines indicate the 95% confidence interval of the regression. Boxplot as in Figure 1. ACT = anticyclonic circulation type.

Intuitively, drought events are expected to occur during time periods when dry CTs are more frequent. Indeed, the frequency anomalies of the CTs during drought events (Figure 2a) change from positive to negative, from dry to wet CTs. D1 shows a median anomaly of 1.5, indicating a 50% frequency increase from the mean, in contrast to W1 and W2, which show an about 30% decrease. The spread of the boxplots is rather large, suggesting that some drought events also have a below average frequency of dry CTs. The frequency anomalies of ACTs (D1, D2, and D3) of the drought events are significantly correlated with the RR deficits (Figure 2b; *F* test of linear regression with *p* < 0.01), and a linear regression model explains 38% of the variance of the RR deficit (see Table 3, first row).

**Table 3:** Explained Variances of Mean Precipitation Deficit by Different Predictors (Frequency Anomaly of ACTs, NAO, EAWR, and AMO) and Predictor Combinations on an Event Basis and on a Seasonal Basis (Subsets of Events, Which Cover Respective Seasons Entirely are Used)

Predictor	Season				
	Winter	Spring	summer	autumn	Event
Frequency anomaly of ACTs	0.50**	0.66**	0.15	0.37**	0.38**
NAO	0.13	0.00	0.12	0.02	0.00
EAWR	0.51**	0.57**	0.16	0.24*	0.16**
AMO	0.01	0.01	0.14	0.06	0.00
Frequency anomaly of ACTs and NAO	0.57**	0.70**	0.27	0.40**	0.41**
Frequency anomaly of ACTs and EAWR	0.63**	0.76**	0.27	0.38**	0.40**
Frequency anomaly of ACTs and AMO	0.65**	0.80**	0.45	0.41*	0.44**
EAWR and NAO	0.56**	0.51**	0.28	0.24	0.05
EAWR and AMO	0.58**	0.50**	0.19	0.26	0.10
NAO and AMO	0.19	0.26	0.15	0.18	0.03
Frequency anomaly of ACTs, EAWR, and NAO	0.65**	0.82**	0.40	0.40*	0.40**
Frequency anomaly of ACTs, EAWR, and AMO	0.75**	0.91**	0.47	0.41	0.47**
Frequency anomaly of ACTs, NAO, and AMO	0.71**	0.81**	0.45	0.41	0.47**
EAWR, NAO, and AMO	0.65**	0.51*	0.30	0.31	0.10
Frequency anomaly of ACTs, EAWR, NAO, and AMO	0.76**	0.92**	0.47	0.41	0.50**

Abbreviations: ACT = anticyclonic circulation type; AMO = Atlantic Multidecadal Oscillation; EAWR = Eastern Atlantic/Western Russia Index; NAO = North Atlantic Oscillation.

* significance at 10% level.

** significance at 5% level.

Table 3 lists the variance of RR deficit (averaged over the duration of the event and over the GAR) explained by regressions with the frequency anomaly of ACTs, the NAO, the EAWR, and the AMO and various combinations of them. No significant collinearity was found among the different indices, as the variance inflation factor is <2 for all variables. Frequency anomaly of ACTs shows the largest explained variances in DJF (0.50) and MAM (0.66) and considerably lower values in SON (0.37) and JJA (0.15). NAO and AMO have very low predictive skill with explained variances ranging between 0.00 and 0.14. In contrast, the EAWR shows values of up to 0.57 (MAM), which is close to the explained variance obtained from the frequency anomaly of ACTs. This may be due to the resemblance of the pressure patterns associated with the positive phase of the EAWR (cf. Ionita, 2014) and the pressure patterns of D1 and D2. On the event basis (i.e., neglecting the season), the explained variance is considerably lower (0.16).

If multiple predictors are considered, the explained variance increases to 0.80 in MAM, using ACT anomaly and AMO as predictors, and even up to 0.92 if all four predictors are used. In DJF these values are somewhat lower (e.g., 0.65 for frequency anomaly of ACTs and AMO, and 0.76 for all four predictors).

In JJA there are no striking relationships with different predictor combinations (0.47 using all four predictors). In SON the explained variances are low as well, but at least two significant relationships arise, one with a combination of ACT anomaly + NAO (explained variance 0.40) and ACT anomaly + EAWR (explained variance 0.38).

These results highlight the importance of circulation anomalies as the main drivers of RR deficit in the cold season, whereas other effects may take over during the warm season, particularly during JJA.

CTs, as derived over the GAR in this case, are generally not independent of the large‐scale atmospheric variability patterns. For instance, CTs D3 and N2 depict a likewise NAO+ phase with westerly flow and meridional pressure gradients, whereas CT D2 is like an EAWR+ pattern with high pressure near the British Isles.

**Figure 3:**
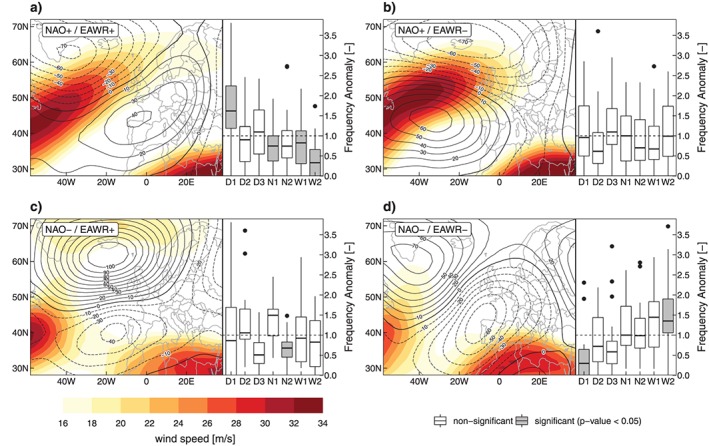
(a–d) Geopotential height at 500‐hPa (z500) anomalies (contours, solid line = positive anomalies, dashed line = negative anomalies) and jet stream wind speeds at 300 hPa (color shading) stratified by combined NAO/EAWR phases; NAO+/EAWR+ (NAO−/EAWR−) indicates index values above (below) 1 standard deviation in winter/spring on the left side of the plot, and respective frequency anomaly distributions of circulation types are displayed as boxplots on the right side; the fill color of the box indicates significant differences (*p* < 0.05) of the NAO/EAWR phase subset compared to the entire sample assessed with the Kolmogorov‐Smirnov test; boxplot as in Figure 1. EAWR = Eastern Atlantic/Western Russia Index; NAO = North Atlantic Oscillation.

To better understand the interrelationships between the CTs, the NAO, and EAWR Figure 3 displays the atmospheric flow conditions (z500 anomalies and jet stream wind speed) as well as respective CT anomalies. Figure 3a shows the conditions for both NAO and EAWR in its positive mode. Due to the positive NAO, prominent meridional pressure gradients emerge over the North Atlantic with a particular active jet stream region. The prevailing flow is from Southwest over the British Isles toward Scandinavia, and positive Geopotential height at 500‐hPa (z500) anomalies emerge in Southwestern and Central Europe. These patterns are associated with wet conditions in Northern Europe, whereas Central Europe (including the GAR) and the Mediterranean are usually dry.

This is also reflected by the frequency anomalies of CTs, where we see significant positive anomalies of D1 as well as slightly but still significant negative anomalies of N1 and W1 and rather importantly, strong negative anomalies of W2, the wettest CT. On the other hand, if the NAO is in its positive phase and the EAWR is negative (Figure 3b), the prevailing flow is predominantly westerly over West Europe with a strong jet stream over the North Atlantic and the z500 anomalies being negative over Central Europe. The reason for this pattern is the negative phase of the EAWR, which is associated with negative pressure anomalies around the Eastern Atlantic, favoring the westerly flow. These subtle differences have profound implications for large‐scale precipitation in the GAR. The frequency anomalies of CTs show no significant deviations, indicating a large variety of the synoptic situations over the Alpine Region during this large‐scale phase configuration. The modulation of the direction of the jet stream downstream over West Europe during NAO+ is obviously modulated by the phase of the EAWR and thus drives the cyclonic activity in Central Europe, determining dry or non‐dry conditions during similarly positive NAO phases.

Negative NAO and positive EAWR conditions (Figure 3c) are associated with an attenuated zonal flow over Europe, positive z500 anomalies over the North Atlantic, and blocking‐like conditions over Northwestern Europe. As illustrated by Ionita (2014), the jet stream splits up during these situations over the North Atlantic and merges toward the South with the subtropical jet and the Northern streak being far deflected toward Iceland and the Norwegian Sea. This enhanced meridional flow and absence of jet stream activity is well related to dry conditions in much of Europe and the GAR (Ionita, 2014). Finally, if both circulation modes are in their negative phase, meridional flow in combination with cyclonic activity over Western and Central Europe prevails (significant negative anomalies of D1 and positive anomalies of W2), leading to wet conditions in this respect.

### 4.2 Drought Driver #2: Precipitation Efficiency

#### 4.2.1 Event Specific Characteristics

As demonstrated above, anticyclonic circulation anomalies explain much of the variability of precipitation deficit during the drought events. However, this is only the case in DJF and MAM. In SON and JJA, other drivers are more important.

We therefore hypothesize that the combined effect of a circulation anomaly in combination with reduced precipitation efficiency is driving an observed RR deficit. It could either result from a positive frequency anomaly of ACTs alone with wet CTs, however bringing sufficient precipitation but being outnumbered by the precipitation deficit of ACTs (RR efficiency ~1). Or it could occur at normal ACT conditions when wet CTs bring insufficient precipitation (RR efficiency <1). A RR deficit is therefore larger, the larger the spread between frequency anomaly of ACTs (>1) and RR efficiency (<1).

**Figure 4:**
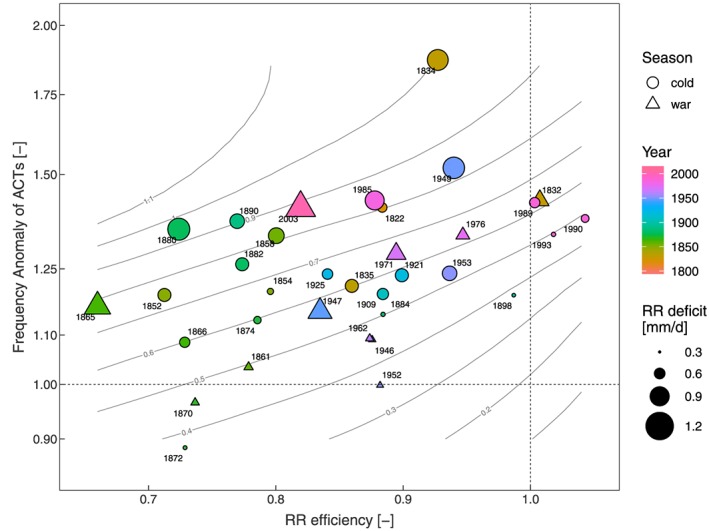
Event‐specific RR efficiency plotted against frequency anomaly of ACTs, the patch symbol represents the respective season (circles—cold season triangles—warm season); the patch color indicates the year of occurrence, and the size of the patch reflects the RR deficit; the gray contours indicate the relationship between RR deficit, RR efficiency, and frequency anomaly of ACTs through a two‐dimensional locally estimated scatterplot smoothing fit. ACT = anticyclonic circulation type; RR = average precipitation.

Figure 4 shows a scatterplot of RR efficiency against frequency anomaly of ACTs where the RR deficit is given by the size of the patch; the colors and labels indicate the year of occurrence, and the patch shape separates warm season from cold season events (see section 3 for details).

During recent decades (1970‐2009), seven events were observed (pink to purple color), four in the cold season and three in the warm season. The cold season events (1985, 1989, 1990, and 1993) are characterized by frequency anomalies of ACTs above 1.25 (mean frequency anomaly of ACTs 1.39) and a mean precipitation efficiency of 0.99. This means that these events are driven mainly by circulation anomalies. For events in the warm season (1971, 1976, and 2003), the mean frequency anomaly of ACTs is 1.34 and the mean precipitation efficiency is 0.88, which is considerably lower than the respective values of the cold season events. Due to the convective characteristics of JJA time precipitation formation, local conditions of moisture sources (soil) are of higher importance, which in turn leads to lower precipitation efficiency if soil moisture states are already low from preceding MAM (Koster et al., 2017; van der Linden et al., 2018). This process was of particular importance in the 2003 event, which started in late DJF, exhibited positive frequency anomalies of ACTs during MAM (1.94 in MAM) leading to soil moisture deficits in early JJA and then through a soil moisture atmosphere coupling to considerably low precipitation efficiency (0.54 in JJA).

Before 1970, similar patterns emerge. The events during the 1940s and 1950s (blue colors) show lower precipitation efficiencies than average (< 1) during warm season events (1946, 1947, 1952, and 1962) and higher frequency anomalies of ACTs than average (> 1) during cold season events (1949 and 1953). The second half of the 19th century (1851–1900, green colors), however, is characterized by substantially lower RR efficiencies for both warm and cold season events (mean precipitation efficiency of 0.78 for 1851–1900). The frequency anomalies of ACTs vary substantially from less than 0.9 to almost 1.5. In the 1860s, frequency anomalies of ACTs were particularly low; two events had even fewer ACTs than expected from the climatology (1870 and 1872). The RR efficiencies in this time period were the lowest in the entire data set. Interestingly, these strong anomalies occur in both seasons, which implies that this finding does not support the above hypothesis of higher precipitation efficiency anomalies during warm season events being due to a soil moisture‐precipitation coupling.

#### 4.2.2 Coupling With Soil Moisture

**Figure 5:**
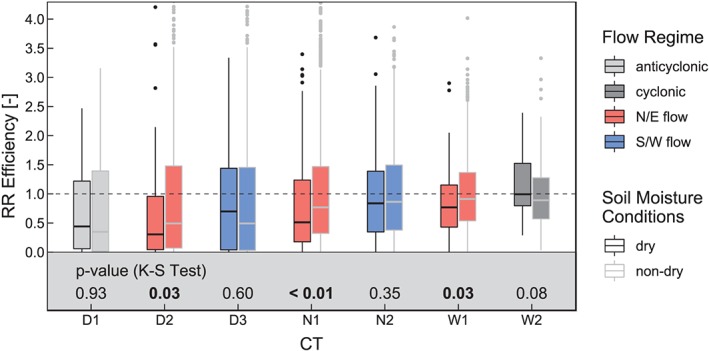
Daily precipitation efficiency in summer stratified by CT using those days when the preceding (one day before the actual day) accumulated 90‐day climatic water balance was below the 10th percentile (dry soil moisture conditions, black box, and whiskers) or above the 90th percentile (non‐dry soil moisture conditions, gray box, and whiskers). Significance of the difference between preceding dry and non‐dry RR efficiency distributions is assessed with the K‐S test; the color coding of the boxplots follows the classification of the flow regime in Table 2. Boxplot as in Figure 1. CT = circulation type; K‐S = Kolmogorov‐Smirnov test.

In order to better understand this coupling we analyzed the precipitation efficiency in JJA as a function of the circulation characteristics and the soil moisture conditions. The precipitation efficiency of the subsequent day under dry and non‐dry conditions (see section 3.2 for details) is displayed in Figure 5, stratified by CT.

The lowest RR efficiencies occur for D1 although it is not very frequent in JJA (see Figure 1), so this estimate has to be treated with caution. However, significant (5% significance level) differences of RR efficiency between dry and non‐dry conditions are apparent for D2 (*p* = 0.03), N1 (<0.01), and W1 (0.03). These distinctions are associated with clearly lower RR efficiencies during dry conditions with a mean RR efficiency of D2 of 0.65 and N1 and W1 of 0.84. In contrast, no significant differences of the RR efficiency distribution are detected for D3 (*p* = 0.60), N2 (0.35), and W2 (0.08). CTs D3 and N2 are circulation patterns where large‐scale zonal flow with distinct pressure gradients allows large‐scale moisture transport from the Atlantic. On the other hand D2, N1, and W1 show a flatter pressure distribution, less zonal, and enhanced meridional flow. The significant differences in RR efficiency with respect to soil moisture conditions are a strong indication for these CTs being sensitive to preceding soil moisture conditions and therefore favoring local moist convection as well.

#### 4.2.3 Comparison of JJA Droughts 1962 and 2003

The 2003 event is in many respects a benchmark event (Laaha et al., 2017) for future climate change conditions, as we expect it was primarily driven by reduced precipitation efficiency due to a negative soil moisture‐precipitation feedback (Black et al., 2004; Fink et al., 2004). However, as reported by HB17, the summer of 2003 was topped by the summer of 1962 in terms of RR deficit, with the remarkable difference that 1962 was cooler than average (−0.4 °C). From a process perspective, the soil moisture precipitation feedback is tied to an increasing sensible heat flux over time at the expense of latent heat flux, forcing temperatures to rise and moist convection to be suppressed (Seneviratne et al., 2010). But this was not the case in the JJA of 1962. Table 4 shows a summary of RR, RR efficiency, and temperature stratified by CTs for both JJAs. The precipitation efficiency for D2 for example in 2003 was much lower (0.08) than that in 1962 (0.28), which is also the case for W1 (1962: 0.81 and 2003: 0.69). In contrast, the CTs less sensitive to soil moisture precipitation feedbacks exhibit lower RR efficiencies during the 1962 JJA, for example, 0.21 for D3 (2003: 0.72) and 0.68 for W2 (2003: 0.92). However, as an exception, this is not the case for N2, where RR efficiency in 1962 (0.62) is higher than that in 2003 (0.37).

**Table 4:** The Summers of 1962 and 2003 in Comparison (Precipitation, Precipitation Efficiency, and Temperature) Stratified by CT

CT	D2 (E)		D3 (W)		N1 (NE)		N2 (WSW)		W1 (N)		W2 (WC)	
Year	1962	2003	1962	2003	1962	2003	1962	2003	1962	2003	1962	2003
Precipitation (mm/d)	0.4	0.1	0.3	0.6	1.2	1.2	2.2	1.5	4.8	4.1	5.1	7.3
RR efficiency	0.28	0.08	0.21	0.72	0.42	0.47	0.62	0.37	0.81	0.69	0.68	0.92
Temperature (°C)	18.3	21.8	18.7	23.4	16.6	20.7	19.7	21.3	16.1	20.3	17.3	20.9

Abbreviations: CT = circulation type; RR = precipitation.

**Figure 6:**
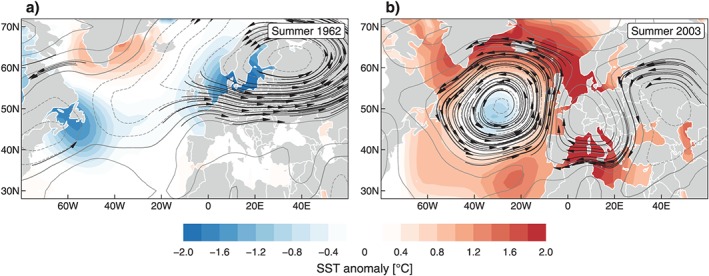
SST anomalies (color shading), geopotential height anomalies at 500‐hPa (contours, 5‐hPa interval), and 500‐hPa wind speeds (arrows) during the summer of (a) 1962 and (b) 2003. SST = sea surface temperature.

These differences are related to the atmospheric circulation and regional SST anomaly patterns as illustrated in Figure 6. In 1962 (Figure 6a) a dominant westerly flow approached Central Europe counteracting the weak high pressure anomalies in Southern Europe and the Mediterranean. In contrast, JJA 2003 shows an extensive high pressure system over Western and Central Europe, blocking the moisture supply from the Atlantic and therefore driving the local soil moisture precipitation feedback. The SST anomalies were strongly positive over vast areas across the North Atlantic, except for the area of persistent cyclonic activity south of Greenland. The SST patterns were quite different in 1962. The North Atlantic was rather cool, and in particular the North and Baltic Seas showed strong negative anomalies. Such patterns seem to significantly influence the precipitation efficiency of D3, N2, and W2, as less moisture is transported from these source regions due to cooler atmospheric and oceanic conditions. Comparing the precipitation efficiency of W2 dependent on warm (+1 °C anomaly) versus cold (−1 °C anomaly) conditions in the Atlantic and the North Sea (20° W–10° E and 40° N–60° N) revealed a mean precipitation efficiency of 1.01 for warm SSTs and 0.72 for cool SSTs, highlighting the importance not only of local soil moisture precipitation feedback altering precipitation efficiency but also of large‐scale SST patterns.

### 4.3 Similarities and Differences of the Drought Decades of the 1860s and 1940s

In order to get a better understanding of the general precipitation characteristics we shift our focus from the detected drought events to a broader view of the entire precipitation distribution (see section 3.3 for details).

**Figure 7:**
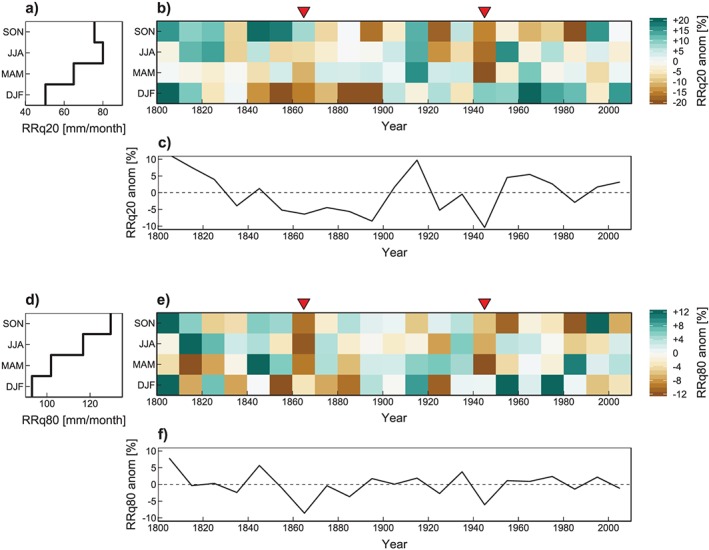
(a) Long‐term (1801–2010) monthly 0.2 quantile (dry tail) precipitation stratified by season, (b) anomalies (wrt. 1801–2010) of the decadal monthly 0.2 quantile precipitation stratified by season and (c) for the entire year. Analogous plots for the 0.8 quantile (wet tail; d,e,f). All estimates are averages over the GAR. Red triangles indicate outstanding drought decades. DJF = winter; JJA = summer; MAM = spring; SON = autumn.

Figure 7a shows the seasonal distribution of the long‐term 0.2 quantile (RRq20, dry tail). The graph points to a minimum in DJF and a maximum in JJA, as would be expected, as rainfall in the GAR is JJA dominated (Parajka et al., 2009). The respective seasonal anomalies per decade of RRq20 (Figure 7b) show that the DJF of the 1860s and 1940s were not the driest on record; however, the dry tail in the 1860s was drier than average, whereas in the 1940s it was even wetter. In contrast, the MAM anomalies are rather pronounced, particularly in the 1940s, which is also the case for the JJA and SON anomalies. On average over the seasons (Figure 7c), the 1940s were exceptional in terms of RRq20 anomalies, but the 1860s less so.

The wet tail of the distribution (0.8 precipitation quantile, RRq80) in Figure 7d shows gradually increasing precipitation from DJF to SON. The anomalies (Figure 7e) are characterized by high fluctuations during the DJF, but the strongest anomalies do not occur the 1860s and 1940s. However, in MAM negative anomalies are pronounced in the 1860s and particularly large in the 1940s. The JJAs are similar, although the signal in the 1860s was much stronger than that in the 1940s. This result is in line with findings of HB17 who found drought events with longer durations during the 1860s, which is a consequence of the absence of extraordinarily wet months. This is not the case for the 1940s events, which show, on average, shorter durations through event breakups caused by extraordinarily wet months.

**Figure 8:**
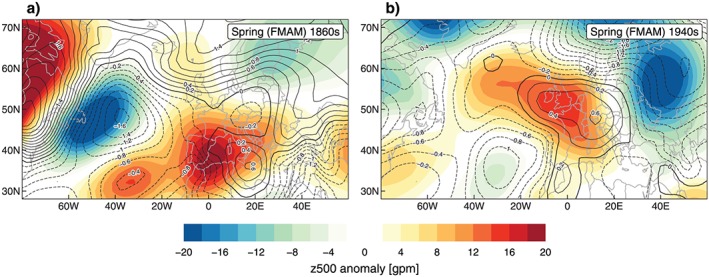
Geopotential height at 500‐hPa anomaly (color shading) and mean sea level pressure anomaly (contours) over Europe and the North Atlantic in FMAM with respect to the long‐term mean (1851–2010) for (a) the 1860s (1861–1875) and (b) the 1940s (1941–1955). FMAM = extended spring.

This analysis indicates MAM as the key season for understanding the emergence of outstanding drought decades as this season showed the strongest anomalies throughout the last 200 years for both the wet and dry tail quantiles. General circulation characteristics are displayed in Figure 8 as of the 500‐hPa geopotential height anomalies (z500, mid troposphere) and the mean sea level pressure anomalies (surface level) during extended MAMs of the 1860s (1861–1875; Figure 8a) and the 1940s (1941–1955; Figure 8b). February is added to the MAM season because the described effects are already present at the transition from DJF to MAM, and the signals are more distinct if an extended MAM season is considered. First of all, the two pressure patterns look quite different, implying rather different atmospheric driving mechanisms. The 1860s are characterized by weak upper level blocking in the Norwegian Sea and generally positive z500 anomalies in Southwestern Europe, contrasting with two negative anomaly areas south of Greenland and over Scandinavia. As can be seen in Figure 4, events during the 1860s show a weak circulation forcing; the frequency anomalies of ACTs are relatively low (1.04 average), some events show even less ACTs compared to the climatology. This is confirmed by the negative pressure anomalies over the GAR (Figure 8a).

**Figure 9:**
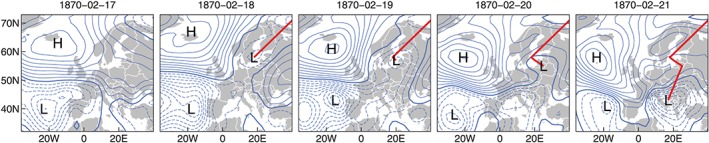
Daily sequence of mean sea level pressure over Europe and the North Atlantic (February 17 to 21, 1870), the main H and L pressure systems and the track of a low pressure system entering Central Europe from the Northeast (red). H = high; L = low.

Additional analyses (not shown) of frequency anomalies of CTs suggest that the 1860s MAMs were characterized by frequent and persistent cyclonic CTs (W1 and W2). This counterintuitive behavior might be explained by a recurring circulation regime as illustrated by a daily sequence (Febuary 17 to 21, 1870) of sea level pressure maps in Figure 9. The circulation is characterized by a strong blocking over Iceland and cyclonic activity over the Azores, resembling a negative NAO mode, which is very similar to the mean state during the 1860s (see Figure 8a). On the second day of the sequence (February 18) a low pressure system emerged over Scandinavia. The formation of the cyclone in that particular area is also referred to as a “diving cyclone” (Semenova & Sumak, 2017). After formation it is propagating southwards toward the GAR during the following days with its center reaching the Adriatic Sea on February 21. Particularly, the latter half of this cyclone track is rather uncommon given its area of generation and track evolution. In a recent analysis, Hofstätter et al. (2017) showed that these cyclone track types are rare, and precipitation totals in Central Europe asociated with these tracks are lower than those of the other track types. Other examples of these circulation characteristics were identified by visual inspection of the sea level pressure fields during droughts in MAM in the 1860s (not shown), which suggest that these are responsible for dry conditions even tough cyclonic activity is present.

The anomalous pressure patterns as shown in Figure 9 could be due to surface‐atmosphere feedback processes in the higher latitude North Atlantic. Reconstructions of sea ice concentrations in the North Atlantic show a distinct peak of maximum sea ice extent during the 1860s and 1870s (Lamb, 1995; Macias Fauria et al., 2010; Vinje, 2001). Corresponding to the positive sea level pressure anomalies north of the British Isles (Figure 9a) these anomalies might be caused by the large‐scale sea ice concentration during late DJF/early MAM, inducing a thermal high at the surface and weak blocking in the mid troposphere (500 hPa).

Compared to the 1860s (Figure 8a), the MAM atmospheric circulation characteristics of the 1940s (Figure 8b) differ significantly. The 1940s exhibit a pronounced positive pressure anomaly both at the surface and the upper level stretching from Ireland toward Southwestern and Central Europe, which strongly resembles a positive EAWR mode. This dominant anticyclonic and therefore likely dry situation is also confirmed by the large negative anomalies of the dry tail precipitation quantile (Figure 7b and 7c).

**Figure 10:**
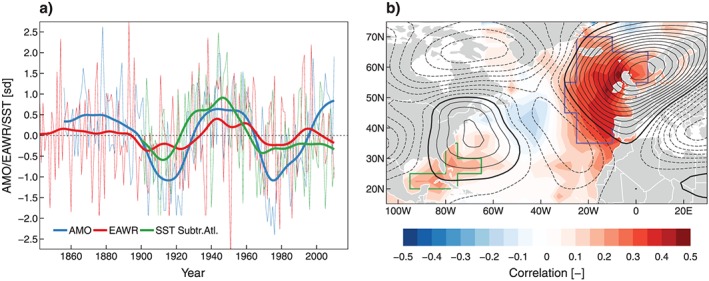
(a) Time series of average late winter/spring standardized AMO index (blue), the EAWR index (red), the detrended SST in the western subtropical Atlantic (60–80 W and 20–40 N, green), and 30‐year Gaussian filtered time series (thick lines); (b) correlation between the EAWR and SST (color shaded areas) and 500‐hPa geopotential height anomaly during the positive (>0) phase of the EAWR (contours), the bounding polygons indicate significant (*p* < 0.05) correlation; the colors of the hatches (green and blue) indicate different causalities of the correlations (see text for details). AMO = Atlantic Multidecadal Oscillation; EAWR = Eastern Atlantic/Western Russia Pattern ; SST = sea surface temperature.

Analyzing the EAWR in MAM (Figure 10a) reveals an expectedly local maximum of the positive phase during the 1940s with the multidecadal evolutions of both the EAWR and the AMO (30‐year Gaussian filter, thick lines in Figure 10a), which seem similar. During 1900–1920 and 1970–1980 both indices are in negative phases, and during 1940–1960 they are in positive phases. The evolution of the EAWR seems therefore tied to SST in the North Atlantic, consistent with the association of cold North Atlantic SSTs and an enhanced Siberian High (and therefore a negative EAWR pattern) found by Wang et al. (2011).

Using a stationary wave model, Lim (2015) showed that positive SST anomalies in the western subtropical Atlantic result in diabatic heating in the mid troposphere, which in turn results in a Rossby‐type wave that resembles the features of the EAWR. This causal chain is in good agreement with the time series of the EAWR (red) and the detrended SST in the subtropical western Atlantic (green) both peaking in the 1940s (Figure. 10a). EAWR and the SSTs are significantly correlated in the subtropical western Atlantic (Figure 10b), which is in good agreement with Lim (2015), and also in the midlatitude eastern Atlantic. However, these two regions differ in terms of the mechanisms behind the correlations. As shown by Lim (2015), the positive SSTs in the western subtropical Atlantic and vorticity transients near the Atlantic jet region (~40°N, ~40°W) are the forcings for a positive EAWR pattern, but other regions in the North Atlantic like east of 30°W and north of 40°N are clearly no forcing region, which indicates that the strong positive correlations between the EAWR and SSTs in Figure 10b (gray hatching) is a response to high pressure over Northwestern Europe‐induced prevailing southerly winds.

**Figure 11:**
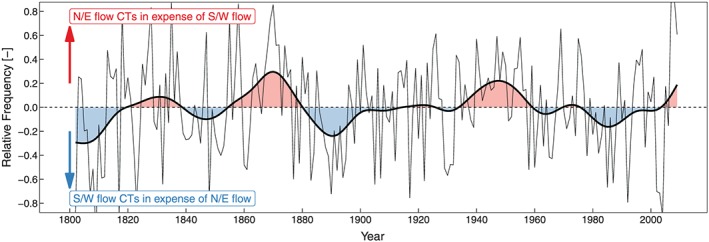
Time series of summer relative frequency difference between north and easterly flows CTs (N1, D2, and W1), south and westerly flow CTs (N2 and D3; thin black line), and 30‐year Gaussian filter (thick black line). Positive frequencies indicate enhanced N/E flows at the expense of S/W flows; negative frequencies indicate enhanced S/W flows. CT = circulation type.

In the JJA season there is a similar signal as in MAM, with the wet tail anomaly being more important in the 1860s and the dry tail anomaly more important in the 1940s (Figure 7b and 7e). We also found that positive frequency anomalies of ACTs are not able to explain much of the observed RR deficit variability during droughts in JJA (Table 3); however, certain CTs are more sensitive to preceding soil moisture conditions than others (Figure 5). A comparison of the relative frequencies of the sensitive CTs (D2, N1, and W1; N/E flow) with the less sensitive CTs (D3 and N2; S/W flow) during JJA is given in Figure 11. The 1860s and 1940s show a pronounced signal of an enhanced frequency of N/E flow CTs at the expense of S/W flow CTs. This means that the general flow conditions are dominated by CTs that are characterized by flatter pressure gradients, potentially allowing for local convection, which in turn implies less large‐scale advection and thus less moisture transport from the Atlantic as a main moisture source. These atmospheric circulation conditions in combination with low soil moisture conditions from the preceding MAM might drive a positive feedback, which allows for a substantial build‐up of precipitation deficit.

## 5 Discussion

### 5.1 General Remarks

For assessing the drivers behind the major drought events occurring in the GAR during the last 210 years we distinguish between atmospheric circulation and precipitation efficiency. Considering driver #1 (atmospheric circulation), we found that positive frequency anomalies of ACTs (enhanced anticyclonic activity over Central Europe) are, not surprisingly, a main driver of drought conditions as noted by Kingston et al. (2015) and Trnka et al. (2009). However, in DJF and MAM the explained variance is even higher, whereas there is no significant correlation in JJA. These findings are consistent with other studies (e.g., Hannaford et al., 2011) that are based on large‐scale atmospheric indicators such as NAO.

The joint analysis of the frequency anomalies of ACTs and dominant large‐scale atmospheric flow suggests that there is no correlation between the NAO and average rainfall deficit in any season, but there is a strong correlation with the EAWR in DJF and MAM. As noted by Ionita (2014) and Kingston et al. (2015), the EAWR pattern, which is a zonal pressure pattern showing positive pressure anomalies in NW Europe during its positive phase, is related to droughts in Europe, in line with our findings for the GAR in DJF and MAM. Other authors have suggested that the NAO is an important driver of European scale droughts (Hannaford et al., 2011; López‐Moreno & Vicente‐Serrano, 2008); however, this relationship does not unfold for the GAR.

We also found that driver #2 (RR efficiency) has a significant effect on precipitation deficit build‐up. We hypothesize that precipitation efficiency is lower in JJA if preceding soil moisture conditions are low and the atmospheric circulation shows weak pressure gradients enabling the formation of local moist convection and rain.

Our results confirm this hypothesis in two ways, first through the generally lower RR efficiency for CTs allowing for the formation of local convective precipitation (cf. Figure 5) and secondly because RR efficiency is found to be lower in the warm season as compared to the cold season in the recent 40 years. Although soil moisture‐precipitation feedbacks are not straightforward to identify (Koster et al., 2017; Tuttle & Salvucci, 2017), our findings are in line with other studies (Findell et al., 2011; Guillod et al., 2015) who found evidence for a positive feedback during the warm season as well. Considering moisture sources for the Alpine region in particular, using a Lagrangian moisture source diagnostics model, Sodemann and Zubler (2009) found that there are distinct differences between seasons, with DJF precipitation being mostly driven by large‐scale advection (continental moisture recycling <10%) and JJA precipitation showing considerably higher continental recycling rates of up to 50%, highlighting the importance of soil moisture precipitation feedbacks. However, as the two driest JJAs during the last 210 years (1962 and 2003) show (Figure 6), the soil moisture precipitation feedback is not necessarily the only driver modulating RR efficiency. Pronounced negative SST anomalies in the Eastern North Atlantic and the North Sea are most likely the reason for reduced RR efficiency in the JJA of 1962, particularly for the CTs associated with North/Western advection due to reduced atmospheric moisture content. The North Sea was rather cool in JJA of 1962, showing the second largest negative SST anomalies between 1950 and 2010, which highlights the peculiarity of this JJA drought.

Our current study also shows that the outstanding drought decade of the 1940s appears not unusual in terms of the drivers when compared to most other droughts during the last 210 years. The time period from 1940 to 1955 was dominated by a strongly positive anomalies of the EAWR in the late DJFs and MAMs. These were followed during the JJAs by positive frequency anomalies of CTs that are sensitive to soil moisture‐precipitation feedbacks, resulting in an enhanced frequency of droughts during that period. Contrary, the processes driving the droughts of the 1860s are not as straightforward to understand. The most counterintuitive aspect is that frequency anomalies of ACTs show no strong indication of enhanced anticyclonic circulation. Moreover, some events show even less ACTs than the climatological mean. This signal is strongest during MAMs, when unusual cyclone tracks move toward the GAR from the Northeast. Recent studies relating precipitation amounts to cyclone tracks (Hofstätter et al., 2016; Hofstätter et al., 2018) in the Alpine region found that this kind of tracks has been rather rare during recent decades and that the associated precipitation has been extremely low. The global climate during the 1860s marked the end of the Little Ice Age, which was rather different from the recent decades. It was a cold period throughout the Northern Hemisphere, with vast sea ice formation in the North Atlantic (Lamb, 1995; Vinje, 2001). The strongly positive sea level pressure anomalies during the MAMs of the 1860s in the North Atlantic found here may in fact have been due to sea ice formation altering the surface energy balance and inducing a thermal high near the surface. These anomalies reaching to the upper level (500 hPa) troposphere have likely blocked the westerly flows and therefore initiated the southwestward propagation of cyclones from the low pressure area over Scandinavia and Western Russia. Coupled atmosphere‐ocean models could be used to further examine these potential mechanisms in future studies, although the ability of such models to reproduce the natural variability of Arctic sea ice extent has to be evaluated.

In addition, the analysis of the droughts in the 1860s also points toward some limitations of the methods applied in this paper related to the concept of precipitation efficiency. The concept is likely applicable for the time period when the mean CT precipitation was estimated from E‐OBS data, since the comparison between frequency anomalies of ACTs and RR efficiency showed higher (lower) impact of the circulation in the cold (warm) season. But when going further back in time toward the 19th century equals a departure from the recent climate to a rather different climate (cooler), which comes along with a diversification of processes controlling RR efficiency through altered atmospheric circulation dynamics (e.g., exceptionally low precipitation efficiency during Little Ice Age peak in the 1860s). The second limitation is the static concept of CT classifications. which did not account for the antecedent atmospheric conditions, and thus the dynamics, of the circulation. The 1860s showed the lowest precipitation efficiency in the last 210 years, most likely due to the large number of cyclones approaching the GAR from the Northeast and thus advecting little moisture. However, these features are not accounted for in the present CT classification (CAP7) since it classifies a given pressure pattern but does not account for the previous conditions. Previous patterns could be integrated, but most common schemes do not account for a time‐dependent classification (Philipp et al., 2010). These findings have profound implications for climate reconstructions in general. Most of the methods assume a stationary relationship between climate and proxy and also for the use of CTs for reconstructing precipitation further back in time. The relationship between CT in a given month and the mean precipitation identified in recent times may not be applicable to the more distant past when different climate states (different hemispheric temperature distribution, sea ice extent, jet stream location, etc.) occurred. Recently, ensemble techniques have been introduced for statistical analogue downscaling, which overcome these issues to some extent (Caillouet et al., 2016; Kuentz et al., 2015). However, they crucially depend on the length of the archive period for deriving past analogues.

### 5.2 Implications for Understanding Future Climate Change

As pointed out by Mishra and Singh (2010), it is very important to understand historical droughts on regional scales to be able to better assess future drought processes in the wake of global climate change, since drought projections are associated with considerable uncertainty (see Haslinger et al., 2016 for the GAR). Over the last two centuries no trends in meteorological droughts (precipitation deficits) have been observed in the GAR (Haslinger & Blöschl, 2017), although soil moisture droughts have increased due to enhanced potential evapotranspiration associated with increasing temperatures, solar radiation, and vegetation activity (Duethmann & Blöschl, 2018; van der Schrier et al., 2007). The results of this paper suggest that the 1860s are not likely to occur in the near future, as they were closely related to the cool climate at the end of the Little Ice Age around 1850 (Matthews & Briffa, 2005) resulting in strong anomalies of the atmospheric circulation, particularly in MAM.

On the other hand, the 1940s drought was forced by a strong positive EAWR pattern in MAM with its origin in positive SST anomalies in the subtropical western Atlantic and subsequent positive frequency anomalies in the JJAs of CTs, which are sensitive to soil moisture precipitation feedbacks. These mechanisms could likely happen in the near future, if ocean and atmospheric circulation dynamics favor enhanced warming in the western subtropical Atlantic, thus driving a positive EAWR phase. Although the outstanding drought decades of the 1860s and 1940s were rather different in terms of their drivers, both underpin the importance of MAM as the most influential season with regard to decadal‐scale drought conditions. This finding has also been recently highlighted by van der Linden et al. (2018). Moreover, in both outstanding drought decades there was a similar above average frequency of N/E flow CTs in JJA, which are more sensitive to soil moisture‐precipitation feedbacks, at the expense of S/W flow CTs, enhancing the drought signal through reduced precipitation efficiency.

It is not clear whether this JJA circulation regime would have developed regardless of preceding dry MAMs, or if it is a direct consequence of the preceding dry MAMs. The latter would have profound implications for future climate change, since MAMs are expected to become dryer (van der Linden et al., 2018) in Central Europe. On the other hand, Gagen et al. (2016) suggests that the storm track variability during the JJAs of the last millennium and its link to meridional temperature gradients (MTG) over Europe has not been driven by external forcing (e.g., aerosols and greenhouse gasses) but has rather been a result of internal variability. They also identified two MTG extremes in the recent past, one during the 1910s with steep MTGs resulting in wet conditions and one during the 1940s with very weak MTGs triggering the 1940s outstanding drought decade.

## 6 Conclusions

In the present study we investigated the atmospheric drivers of extreme meteorological drought events in the Greater Alpine Region during the past 210 years using a daily atmospheric CT reconstruction tailored to the Alpine region. Our results suggest that positive frequency anomalies of ACTs are the main driver of drought in DJF and MAM, with their occurrence strongly tied to positive EAWR conditions, while the NAO has no significant impact. In JJA a positive soil moisture precipitation feedback is detected, which is strongest when CTs with weak pressure gradients dominate, favoring the development of local convective precipitation. The events of the outstanding dry decades of the 1860s and 1940s were triggered by strong precipitation anomalies during MAM and enhanced through soil moisture precipitation feedbacks during JJA. For the 1860s drought, the dry MAMs were caused by very unusual circulation patterns, which appear to be related to a large Arctic sea extent after the last peak of the Little Ice Age. Differently, the dry MAMs of the 1940s were related to positive SST anomalies in the western subtropical Atlantic, triggering distinct Rossby wave trains leading to persistent positive EAWR circulation patterns. Future research will investigate drought development during the warm season in more detail, particularly the transition from MAM (circulation dominated) to JJA (feedback dominated), since there is evidence of a feedback not only between soil moisture and convective precipitation but also with the atmospheric circulation.
